# Change in Medical Student Preparation for USMLE Step 1 after the Transition to Pass/Fail Reporting: A Retrospective Cohort Study in an International Medical Program

**DOI:** 10.1007/s40670-026-02879-1

**Published:** 2026-09-11

**Authors:** Gerald D Denton, Christian P Gray

**Affiliations:** 1 Ochsner Health / University of Queensland, New Orleans, LA USA; 2 The University of Queensland, Brisbane, QLD Australia

**Keywords:** USMLE Step 1, Medical education, Assessment, Retrieval practice, Self-regulated learning, Pass/fail assessment

## Abstract

In 2022, the USMLE Step 1 transitioned to pass/fail reporting, coinciding with a national decline in first-time pass rate. Study behaviors, particularly engagement with retrieval-based practice, are strong predictors of Step 1 readiness. This study examined changes in study behavior before and after the pass/fail transition at a single institution requiring a passing score on an NBME Comprehensive Basic Science Examination (CBSE) as part of a Step 1 preparation course. This retrospective study analyzed UWorld question bank engagement among second-year medical students at the Ochsner Health/University of Queensland Medical School. First-time Step 1 pass rates declined from 97% to 93% between pre-change (2019–2021) and post-change cohorts (2022–2024). The post-change cohort completed 26% more UWorld questions early in their preparation period but reduced question usage by 36% after achieving a passing CBSE score, whereas pre-change students increased usage in the months following the CBSE prior to sitting Step 1. Reduced retrieval-based study activity following CBSE success may contribute to declining Step 1 performance in the pass/fail era. These findings suggest that readiness assessments may influence subsequent student study behaviors, highlighting the need for medical programs to encourage sustained practice engagement after assessment milestones.

## Introduction

A growing number of countries are implementing national examinations to ensure the training of safe and effective clinicians [1]. In the United States, the United States Medical Licensing Examination (USMLE) is administered by the National Board of Medical Examiners (NBME) as a three-step assessment sequence, with Step 1 typically completed pre-clerkship and focused on assessing foundational biomedical science knowledge [2].

Since 2022, the first-time passing rate for US/Canada MD candidates on Step 1 has fallen from 97% (2019–2021) to 92% (2022–2024) [3]. At our institution, the first-time passing rate has followed a similar trend, decreasing from 97% (2019–2021) to 93% (2022–2024).

The primary explanation for the decrease in the first-time passing rate is the January 2022 increase in the Step 1 passing standard from 194 to 196, which coincided with the transition to pass/fail score reporting [4]. The COVID-19 pandemic also changed educational environments and negatively influenced medical student academic performance [5, 6]. In April 2020, the Association of American Medical Colleges recommended a pause in direct patient contact for medical students [7]. This recommendation led to widespread adoption of virtual and asynchronous learning, replacing in-person learning for preclinical students. An overall decline in academic performance has been reported following the pandemic across multiple levels of education [6, 8].

An additional potential contributor to the lower Step 1 passing rate is a change in student preparation behaviors following the transition to pass/fail reporting. Prior research has identified several preparation behaviors that influence Step 1 performance, including study duration, resource use; and the structure of dedicated study periods. A consistent predictor of Step 1 success is the number of practice questions completed [9–12]. Additionally, NBME Comprehensive Basic Science Examination (CBSE) assessments provide meaningful predictions of Step 1 outcomes [13–15]. However, less is known about how readiness assessment may influence subsequent student behavior in the pass/fail era.

The objective of this study was to examine changes in Step 1 study behaviour before and after the transition to pass/fail score reporting at a single institution that requires completion of a preparatory assessment.

## Methods

**Fig. 1:**
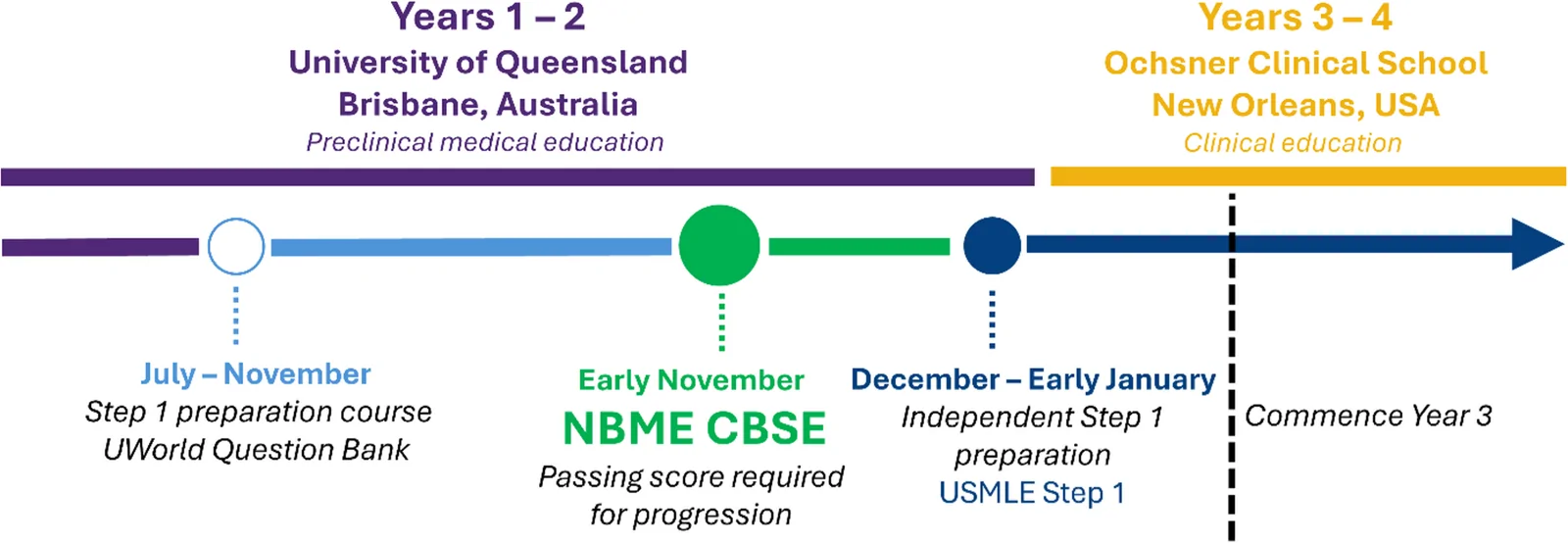
Structure of the Ochsner Health/University of Queensland medical program and timing of USMLE Step 1 preparation and assessment. Students complete two years of preclinical medical education at The University of Queensland in Brisbane, Australia, followed by two years of clinical education at Ochsner Clinical School in New Orleans, USA. During Year 2, students undertake a structured USMLE Step 1 preparation course from July through November using the UWorld question bank. Progression requires achievement of a passing score on the NBME Comprehensive Basic Science Examination (CBSE) in early November. Students subsequently undertake a period of independent Step 1 preparation before sitting the USMLE Step 1 examination in December or early January before commencing Year 3 clinical training

The Ochsner Health/University of Queensland Medical School (OCS) enrolls US citizens and permanent residents for two years of preclinical study in Brisbane, Australia, followed by two years of clinical training in New Orleans, Louisiana. Unlike most US medical schools, OCS operates on an Australian academic calendar, with the academic year aligned to the calendar year (Fig. 1).

During Year 2, students complete a locally developed USMLE Step 1 preparation course delivered from July through November and supported by the UWorld Step 1 question bank. To pass the course, students must achieve a Step 1-equivalent passing score on the NBME Comprehensive Basic Science Examination (CBSE), which is used to assess readiness for the USMLE Step 1 examination. Following successful completion of the CBSE, students undertake a period of independent Step 1 preparation before sitting the examination in December or early January.

A total of 449 students were included in the analysis. The pre-change cohort comprised 256 students (2019–2021), while the post-change cohort comprised 193 students (2022–2024). Students are required to sit the USMLE Step 1 examination in late December or early January to commence their third year of clinical training in New Orleans.

The principal study resource provided is the UWorld Step 1 question bank, a widely used and commonly recommended resource for Step 1 preparation [11]. Because OCS purchases institutional access, faculty are able to track student question on a monthly basis.

This retrospective study compared UWorld question bank usage during the three years prior to the changes to pass/fail Step 1 reporting (2019–2021) with usage during the three years following the transition (2022–2024). Differences in monthly question usage were analyzed using Bonferroni’s multiple comparison test to adjust for repeated comparisons across time points.

## Results

Across both cohorts, 449 students attempted Step 1. In the pre-change cohort (2019–2021), 248 of 256 students (97%) passed on their first attempt, while 8 (3%) failed. In the post-change cohort (2022–2024), 179 of 193 students (93%) passed, while 14 (7%) failed. This represents a decline of approximately 4% in the first-time pass rate between the two cohorts.

**Fig. 2:**
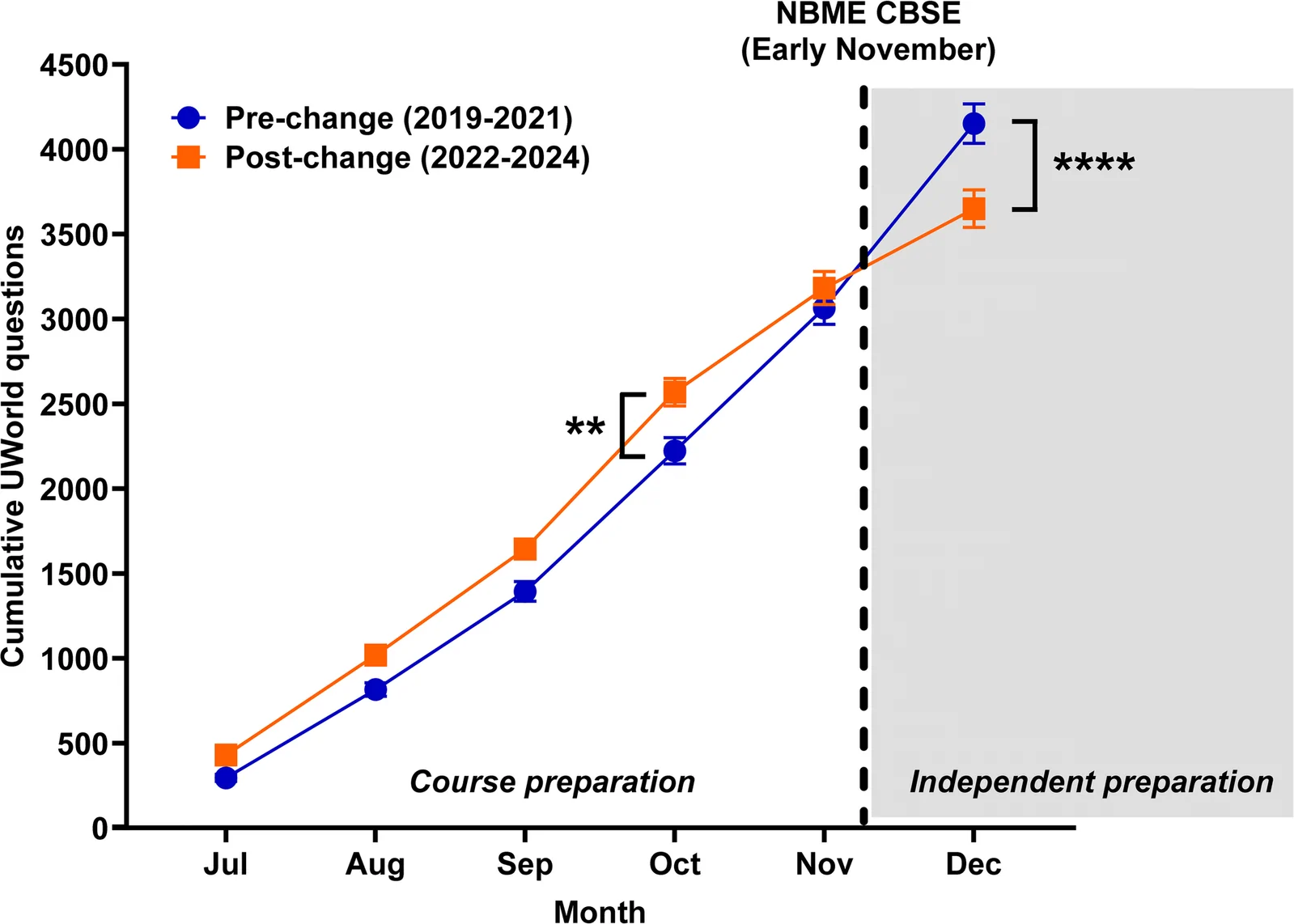
Cumulative UWorld question completion during Step 1 preparation in pre-change and post-change cohorts. Students in the post-change cohort (2022-2024) completed more UWorld questions during the structured preparation course prior to the NBME CBSE than students in the pre-change cohort (2019-2021). After successful completion of the CBSE, students in the pre-change cohort continued to increase question use during the independent preparation period, whereas students in the post-change cohort demonstrated substantially lower engagement. The shaded region represents the post-CBSE independent preparation period. Error bars represent SEM. ** p < 0.01; **** p < 0.0001 (Bonferroni multiple comparison test)

Figure 2 illustrates the cumulative number of UWorld questions completed during the Step 1 preparation period. In both cohorts, the monthly number of UWorld questions completed increased steadily from July through October, prior to CBSE administration in early November. Compared with the pre-change cohort, students in the post-change cohort completed significantly more UWorld questions by October. As shown in Fig. 2, this pattern reversed following completion of the CBSE in early November, with post-change students demonstrating substantially lower engagement during the independent preparation period. Table 1 provides the monthly breakdown of UWorld questions completed by students who passed the CBSE. Compared with the pre-change period, students in the post-change cohort completed 26% more questions by October (2031 vs. 2553; *p* < 0.01, Bonferroni’s multiple comparison test). In the pre-change cohort, question usage remained high after completion of the CBSE in November and increased further in December. In contrast, in the post-change cohort, question usage declined substantially after CBSE completion, decreasing by 36% from November to December (1702 to 1088; *p* < 0.0001, Bonferroni’s multiple comparison test). Total engagement across the six months of preparation was similar between cohorts.

## Discussion

Several factors may explain the decline in Step 1 pass rates observed nationally and at our institution following the transition to pass/fail reporting. The increase in the passing threshold from 194 to 196 and the educational disruption associated with COVID-19 are likely major contributors. Our findings suggest an additional behavioral factor: reduced engagement in retrieval-based study activities after students achieved a passing score on the required readiness assessment.

In the post-change cohort, students decreased their UWorld usage by 36% after passing the CBSE, whereas pre-change students maintained or increased question engagement during the same period. This divergence occurred during a critical consolidation window prior to sitting Step 1 and coincided with a decline in first-time Step 1 pass rates. Prior research consistently demonstrates that the number of practice questions completed is among the strongest predictors of Step 1 success [9–12]. The observed reduction in retrieval-based practice therefore represents a plausible behavioral mechanism contributing to lower performance in the pass/fail era.

Prior to the transition to pass/fail reporting, USMLE Step 1 scores were widely considered an important component of residency application assessment and were frequently used by residency programs as an objective metric for applicant comparison. The transition to pass/fail reporting removed this source of score differentiation and may have altered students’ perceptions of the value of continued score maximization once minimum readiness requirements had been achieved. In this context, a passing CBSE score may increasingly be interpreted as evidence that sufficient preparation has occurred, reducing motivation for continued intensive retrieval-based practice despite the ongoing requirement to successfully pass Step 1. These behavioral changes can be interpreted through established motivational and metacognitive frameworks. According to expectancy-value theory, learners allocate effort based on the perception of the importance and difficulty of a task [16]. The transition to pass/fail reporting may have reduced the perceived utility of continued intensive preparation once minimum progression requirements had been met, particularly after a readiness assessment indicated a high likelihood of success. In addition, Chang et al. demonstrated that while critical thinking and self-regulated learning strategies positively correlate with progress examination performance, overconfident metacognitive judgments correlate negatively [17]. Passing a preparatory assessment may therefore create an “illusion of competence,” leading some students to prematurely reduce study effort despite remaining gaps in conceptual integration [18]. This interpretation is consistent with the observed reduction in retrieval-based practice following CBSE success in the post-change cohort. In the context of a structured Step 1 preparation course that includes a high-stakes CBSE requirement for progression, students may also experience study fatigue or burnout, contributing to a decline in study intensity once this milestone has been achieved [19]. Together, these motivational, metacognitive and affective factors may contribute to declining engagement following assessment success in the pass/fail era, although these hypotheses warrant further investigation. Previous studies have consistently identified the CBSE as a strong longitudinal predictor of Step 1 performance [5, 13–14]. Our findings extend this work by suggesting that readiness assessments may influence not only performance prediction but also subsequent study behavior, particularly in the pass/fail context, where students may adjust their effort based on perceived rather than actual readiness.

This study has limitations. It was conducted at a single institution that operates as a hybrid US/Australian medical program, which may limit generalizability to other educational settings. The academic calendar aligns with the calendar year rather than the traditional US academic schedule, further limiting comparison. Additionally, we did not examine changes in study behavior following initial CBSE non-passing, as CBSE non-passing at our institution is often transient and followed by subsequent re-attempts.

Despite these limitations, this study provides insights into how study behaviors have changed following the transition to pass/fail Step 1 reporting. The observed post-assessment decline in retrieval-based practice represents a meaningful and potentially modifiable behavior. For medical educators, these findings underscore the importance of reinforcing expectations of sustained retrieval practice beyond readiness milestones and supporting students in accurately calibrating their readiness for licensure examinations.

**Table 1:** Average monthly number of UWorld questions completed by students who passed the CBSE, comparing pre-change (2019–2021) and post-change (2022–2024) periods

	Pre-change period	Post-change period	Difference
July	270	435^***^	165
August	476	583^*^	107
September	518	637^*^	119
October	767	898^**^	131
November	737	630	-107
December	965****	458	-507
Total	3733	3641	-92

* *p* < 0.05, ** *p* < 0.01; *** *p* < 0.001; **** *p* < 0.0001 Bonferroni’s multiple comparison test

## Data Availability

The datasets generated and/or analyzed during the current study are not publicly available due to institutional and participant privacy requirements but may be available from the corresponding author on reasonable request and subject to University of Queensland ethics and data governance approvals.
